# Supplementary material for: MEME-LaB: motif analysis in clusters
Source: Bioinformatics. 2013 May 14;29(13):1696–7. doi: 10.1093/bioinformatics/btt248 (PMC3694638; doi:10.1093/bioinformatics/btt248)
Supplement: Supplementary Data [file supp_btt248_Supplementary_Data.zip › Supplementary_Data/Results_Files/which_gene_ids/cluster22_motif1.html]

Cluster 22 Motif 1


Cluster 22 Motif 1 is found in the following promoters

| Gene ID | Strand | Position\* |
| --- | --- | --- |

|  |  |  |
| --- | --- | --- |
| AT1G01960 | - | 294/500 |
| AT1G02140 | + | 235/500 |
| AT1G02740 | + | 459/500 |
| AT1G04170 | + | 160/500 |
| AT1G04300 | - | 482/500 |
| AT1G04555 | - | 98/500 |
| AT1G04690 | - | 124/500 |
| AT1G05380 | - | 102/500 |
| AT1G07360 | + | 401/500 |
| AT1G08120 | - | 178/500 |
| AT1G08750 | - | 285/500 |
| AT1G09730 | + | 167/500 |
| AT1G10240 | - | 31/500 |
| AT1G10270 | - | 484/500 |
| AT1G11330 | + | 405/500 |
| AT1G11960 | + | 352/500 |
| AT1G12060 | + | 487/500 |
| AT1G12930 | + | 421/500 |
| AT1G14510 | + | 437/500 |
| AT1G14650 | + | 309/500 |
| AT1G15240 | - | 70/500 |
| AT1G15440 | - | 396/500 |
| AT1G17590 | - | 153/500 |
| AT1G18190 | + | 326/500 |
| AT1G18260 | + | 57/500 |
| AT1G18880 | - | 100/500 |
| AT1G19580 | + | 77/500 |
| AT1G20580 | + | 93/500 |
| AT1G20693 | + | 363/500 |
| AT1G20770 | + | 114/500 |
| AT1G22300 | + | 257/500 |
| AT1G23130 | - | 278/500 |
| AT1G26900 | + | 326/500 |
| AT1G30000 | + | 383/500 |
| AT1G31812 | + | 338/500 |
| AT1G31814 | + | 70/500 |
| AT1G34780 | - | 433/500 |
| AT1G35220 | - | 488/500 |
| AT1G45000 | - | 39/500 |
| AT1G45160 | - | 164/500 |
| AT1G47250 | - | 427/500 |
| AT1G48790 | + | 343/500 |
| AT1G48830 | - | 178/500 |
| AT1G49710 | + | 130/500 |
| AT1G51510 | + | 393/500 |
| AT1G53040 | + | 257/500 |
| AT1G53345 | - | 383/500 |
| AT1G53750 | + | 276/500 |
| AT1G58380 | - | 133/500 |
| AT1G60530 | + | 68/500 |
| AT1G60900 | - | 301/500 |
| AT1G61670 | - | 478/500 |
| AT1G64385 | + | 279/500 |
| AT1G65440 | - | 400/500 |
| AT1G66345 | - | 191/500 |
| AT1G73180 | - | 454/500 |
| AT1G73450 | + | 8/500 |
| AT1G75010 | - | 482/500 |
| AT1G78770 | - | 280/500 |
| AT1G79020 | + | 140/500 |
| AT1G79340 | - | 276/500 |
| AT1G79490 | - | 315/500 |
| AT1G79930 | + | 442/500 |
| AT2G01820 | - | 398/500 |
| AT2G02150 | - | 382/500 |
| AT2G04630 | + | 389/500 |
| AT2G04740 | + | 211/500 |
| AT2G06530 | + | 485/500 |
| AT2G17670 | + | 84/500 |
| AT2G17790 | - | 475/500 |
| AT2G17820 | - | 424/500 |
| AT2G18245 | - | 482/500 |
| AT2G18465 | - | 158/500 |
| AT2G19540 | + | 94/500 |
| AT2G19790 | + | 489/500 |
| AT2G20190 | + | 115/500 |
| AT2G20310 | - | 435/500 |
| AT2G20330 | + | 327/500 |
| AT2G20400 | + | 315/500 |
| AT2G21160 | - | 488/500 |
| AT2G22430 | + | 382/500 |
| AT2G23930 | + | 377/500 |
| AT2G25870 | + | 154/500 |
| AT2G26060 | - | 275/500 |
| AT2G26460 | + | 67/500 |
| AT2G27110 | + | 456/500 |
| AT2G27460 | + | 371/500 |
| AT2G27600 | - | 289/500 |
| AT2G29020 | - | 259/500 |
| AT2G30860 | + | 246/500 |
| AT2G31280 | + | 256/500 |
| AT2G32410 | - | 469/500 |
| AT2G35100 | + | 428/500 |
| AT2G35900 | + | 468/500 |
| AT2G35940 | - | 31/500 |
| AT2G36340 | + | 371/500 |
| AT2G37020 | - | 408/500 |
| AT2G37195 | - | 464/500 |
| AT2G37550 | + | 373/500 |
| AT2G37680 | + | 357/500 |
| AT2G38185 | + | 331/500 |
| AT2G38670 | - | 404/500 |
| AT2G39960 | - | 310/500 |
| AT2G41960 | + | 119/500 |
| AT2G44820 | + | 280/500 |
| AT2G45280 | + | 15/500 |
| AT2G46000 | + | 181/500 |
| AT2G46280 | + | 352/500 |
| AT2G47210 | - | 260/500 |
| AT2G47330 | + | 380/500 |
| AT2G47610 | - | 364/500 |
| AT2G48100 | + | 396/500 |
| AT2G48160 | - | 481/500 |
| AT3G01340 | + | 76/500 |
| AT3G01720 | + | 215/500 |
| AT3G02600 | - | 482/500 |
| AT3G04650 | + | 420/500 |
| AT3G06310 | + | 253/500 |
| AT3G06480 | - | 386/500 |
| AT3G06650 | + | 156/500 |
| AT3G07170 | + | 301/500 |
| AT3G07940 | + | 186/500 |
| AT3G08947 | - | 458/500 |
| AT3G09090 | + | 6/500 |
| AT3G09630 | - | 111/500 |
| AT3G10120 | + | 371/500 |
| AT3G11250 | + | 290/500 |
| AT3G11910 | + | 80/500 |
| AT3G11950 | + | 173/500 |
| AT3G12280 | - | 379/500 |
| AT3G12640 | + | 329/500 |
| AT3G12670 | - | 337/500 |
| AT3G13235 | + | 79/500 |
| AT3G15095 | + | 230/500 |
| AT3G16050 | - | 361/500 |
| AT3G17300 | + | 252/500 |
| AT3G17450 | + | 340/500 |
| AT3G17900 | + | 369/500 |
| AT3G18380 | + | 272/500 |
| AT3G18780 | + | 295/500 |
| AT3G20290 | + | 1/500 |
| AT3G20630 | - | 74/500 |
| AT3G20780 | - | 272/500 |
| AT3G22320 | + | 288/500 |
| AT3G24200 | - | 489/500 |
| AT3G25120 | + | 299/500 |
| AT3G25680 | - | 199/500 |
| AT3G26115 | - | 167/500 |
| AT3G26370 | + | 452/500 |
| AT3G27080 | - | 133/500 |
| AT3G27670 | - | 356/500 |
| AT3G30380 | - | 78/500 |
| AT3G45630 | + | 300/500 |
| AT3G45890 | + | 443/500 |
| AT3G48900 | - | 470/500 |
| AT3G49350 | - | 489/500 |
| AT3G49870 | + | 19/500 |
| AT3G51260 | - | 169/500 |
| AT3G51770 | + | 396/500 |
| AT3G51780 | + | 386/500 |
| AT3G52100 | + | 438/500 |
| AT3G52890 | + | 26/500 |
| AT3G53870 | - | 329/500 |
| AT3G55140 | - | 318/500 |
| AT3G55320 | + | 411/500 |
| AT3G55350 | - | 487/500 |
| AT3G55460 | - | 489/500 |
| AT3G57950 | + | 443/500 |
| AT3G58640 | + | 325/500 |
| AT3G59990 | - | 424/500 |
| AT3G60400 | + | 454/500 |
| AT3G60520 | - | 483/500 |
| AT3G60830 | - | 488/500 |
| AT3G61690 | + | 439/500 |
| AT3G62080 | - | 91/500 |
| AT3G62370 | + | 65/500 |
| AT3G62840 | + | 388/500 |
| AT4G00570 | - | 114/500 |
| AT4G00630 | - | 233/500 |
| AT4G00660 | + | 372/500 |
| AT4G01860 | - | 95/500 |
| AT4G02570 | + | 414/500 |
| AT4G10030 | + | 319/500 |
| AT4G10270 | + | 35/500 |
| AT4G11640 | - | 41/500 |
| AT4G12590 | + | 478/500 |
| AT4G13640 | - | 231/500 |
| AT4G14330 | + | 7/500 |
| AT4G14716 | - | 175/500 |
| AT4G16070 | - | 486/500 |
| AT4G16150 | - | 41/500 |
| AT4G16430 | + | 410/500 |
| AT4G16440 | - | 215/500 |
| AT4G16490 | - | 485/500 |
| AT4G16720 | + | 192/500 |
| AT4G17910 | - | 162/500 |
| AT4G18530 | + | 278/500 |
| AT4G19540 | - | 452/500 |
| AT4G20980 | - | 415/500 |
| AT4G21670 | + | 259/500 |
| AT4G21710 | - | 243/500 |
| AT4G21800 | - | 248/500 |
| AT4G22140 | - | 410/500 |
| AT4G23230 | + | 423/500 |
| AT4G24470 | + | 419/500 |
| AT4G24560 | + | 465/500 |
| AT4G25210 | + | 481/500 |
| AT4G29790 | + | 182/500 |
| AT4G31080 | + | 129/500 |
| AT4G31580 | - | 69/500 |
| AT4G31600 | - | 36/500 |
| AT4G34450 | + | 97/500 |
| AT4G34670 | - | 217/500 |
| AT4G35910 | + | 272/500 |
| AT4G35987 | + | 472/500 |
| AT4G38170 | - | 231/500 |
| AT4G38740 | + | 113/500 |
| AT4G39240 | + | 234/500 |
| AT4G39520 | + | 61/500 |
| AT4G39860 | - | 125/500 |
| AT4G40050 | - | 310/500 |
| AT5G01290 | + | 35/500 |
| AT5G01970 | - | 236/500 |
| AT5G02870 | - | 97/500 |
| AT5G04940 | + | 374/500 |
| AT5G06910 | + | 391/500 |
| AT5G07350 | - | 465/500 |
| AT5G08550 | - | 358/500 |
| AT5G08580 | + | 123/500 |
| AT5G09400 | + | 293/500 |
| AT5G10070 | + | 298/500 |
| AT5G10710 | + | 364/500 |
| AT5G11030 | + | 385/500 |
| AT5G11900 | + | 22/500 |
| AT5G11980 | - | 46/500 |
| AT5G12290 | + | 100/500 |
| AT5G13030 | + | 373/500 |
| AT5G13850 | - | 364/500 |
| AT5G16210 | - | 354/500 |
| AT5G18190 | + | 487/500 |
| AT5G20290 | - | 300/500 |
| AT5G22760 | - | 172/500 |
| AT5G23900 | + | 298/500 |
| AT5G24740 | - | 424/500 |
| AT5G25150 | - | 84/500 |
| AT5G26710 | + | 454/500 |
| AT5G26830 | - | 473/500 |
| AT5G27270 | + | 487/500 |
| AT5G27320 | - | 157/500 |
| AT5G27850 | + | 312/500 |
| AT5G27990 | - | 169/500 |
| AT5G35080 | + | 211/500 |
| AT5G37350 | + | 155/500 |
| AT5G40300 | - | 469/500 |
| AT5G41320 | - | 408/500 |
| AT5G41360 | + | 381/500 |
| AT5G42920 | + | 457/500 |
| AT5G42950 | + | 275/500 |
| AT5G43900 | - | 227/500 |
| AT5G48580 | - | 308/500 |
| AT5G52880 | - | 202/500 |
| AT5G53140 | + | 136/500 |
| AT5G55130 | + | 64/500 |
| AT5G56290 | - | 30/500 |
| AT5G56630 | + | 392/500 |
| AT5G59180 | - | 77/500 |
| AT5G59830 | + | 313/500 |
| AT5G59980 | - | 361/500 |
| AT5G61060 | - | 66/500 |
| AT5G61840 | + | 290/500 |
| AT5G64070 | + | 452/500 |
| AT5G66880 | - | 318/500 |
| AT5G66950 | + | 102/500 |
| AT5G67530 | - | 58/500 |

**\*** Overlapping sequences are merged to avoid redundancy. As a result some sequences in the final set may be longer than the specified maximum.

Distribution

+

-

Position values represent number of bases from the 5 prime end of the sequence provided to MEME and the length of that sequence. It is these figures expressed as proportions that are used for statistical analysis.

Probability value of comparison of these values to a linear distribution, p = 2.9e-05

The motif is found 150 times on the + stand and 124 times on the - strand.

Probability value that it is evenly distributed between the two strands, p = 0.065

Move the mouse pointer over the gene ids to highlight the corresponding marker
